# Identifying stakeholder priorities in use of wearable cameras for researching parent-child interactions

**DOI:** 10.3389/frcha.2023.1111299

**Published:** 2023-06-23

**Authors:** Andy Skinner, Ilaria Costantini, Chris Stone, James Darios, Mike Gray, Iryna Culpin, Rebecca M. Pearson

**Affiliations:** ^1^ Bristol Medical School, University of Bristol, Bristol, United Kingdom; ^2^ Integrative Cancer Epidemiology Programme, University of Bristol, Bristol, United Kingdom; ^3^ School of Psychological Science, University of Bristol, Bristol, United Kingdom; ^4^ Research and Strategy, Kinneir Dufort, Bristol, United Kingdom; ^5^ Electronics and Software, Kinneir Dufort, Digital Product Design Consultancy, Bristol, United Kingdom; ^6^ Department of Psychology, Manchester Metropolitan University, Manchester, United Kingdom

**Keywords:** wearable camera, priorities, parent, child, interactions

## Abstract

Wearable Cameras (WCs) enable researchers to capture objective descriptions of what participants see and experience as they go about their normal lives. When studying interactions between individuals (e.g. between a parent and child), using multiple WCs can provide highly detailed descriptions of interactions with levels of ecological validity not possible with other methods. However, the use of WCs brings challenges too, and understanding these is key to developing and optimising these methods. We captured the challenges experienced by a variety of stakeholders, namely parents and a range of different researcher roles (academics, field-workers and data processors) involved in a large UK study exploring parent-child interactions using low-cost, off-the-shelf WCs. High among the challenges identified were difficulties caused when subjects are temporarily not in view in the video footage captured. This and other factors identified were used as criteria to select a new, improved WC. The new WCs reduced the time faces were not in view by 75%. We report this and the other challenges identified, and suggest how these can be used to guide and help optimise future studies of this kind.

## Introduction

The last two decades have seen the adoption of wearable cameras (WCs) in a variety of research domains, from the objective measurement of lifestyle behaviours (1), to developing methods for ethnography (2), to tools for enhancing self-management of chronic diseases (3). There are a number of benefits in using WCs devices. The cameras capture what the wearer sees and interacts with as they move through their environment, providing passive, objective measures of environment and behaviours in free-living conditions. The devices also remove the need for a researcher to be present, which is particularly important in maintaining the ecological validity of studies focused on natural interactions between people (see Supplementary Materials for further detail).

One further advantage of WCs is that they capture the scene from the perspective of the individual. This is particularly beneficial when studying children as their height, and the limited mobility of infants, mean they have a very different perspective on the world to adults. This has led researchers to use WCs to explore a number of factors in children, including the frequency and variation in facial expressions and identities infants encounter (4), the broad nature of children's visual environments (5), and children's daily exposure to food marketing (6). This team has adapted features from many of these studies to use WCs to explore links between parent-child interactions and parental depression (7, 8). A unique feature of these studies is that they combine WCs in both parent and child to explore their joint experiences by capturing different perspectives. For example, synchronised footage captured from the parent WC shows the child and the child WC the parent, but also if the parent or child leaves the room the footage captures where they go. A further application of WCs could be as a tool to enhance video feedback used to improve parent-child interactions therapeutically (9).

However, in addition to these benefits, the use of WCs brings a variety of technical, practical and analytical challenges too. In their review of the experiences of adults using WCs to record a variety of health-related behaviours, Wilson and colleagues (10) found participants were initially concerned about cameras being intrusive, but for most participants this was not felt to be the case in practice, while some participants reported that design issues with the camera's user interface had limited their interactions with the cameras. Madison et al.'s scoping review (3) of the use of WCs in disease self-management identified a number of technical challenges, most significantly the difficulty and effort required in identifying specific behaviours in the footage captured.

In terms of the challenges around using WCs with children, Smith and colleagues' review of the use of WCs to study children's environments (5) listed a number of practical challenges, including the need for the camera to have a wide field of view (given the tendency for infants and children to shift focus and turn their heads more frequently), and have methods of fixing that are both secure and well tolerated by the child. Signal et al. (6) identified that coding the images captured from child worn WCs is resource intensive. While coding footage from adult WCs can also take considerable effort, this appears to be exacerbated in footage from young children, again possibly because reduced attention span leads to an increase in head movements which makes the coding of facial attributes more difficult.

Turning to the issues specific to the use of WCs to explore interactions between children and parents, our initial pilot work in parents and children from a UK cohort study (7) again highlighted the importance of secure and correct placement of the WCs, and the intensive nature of coding the footage captured. Further suggestions for improvements to WCs included better controls, and increased battery life and storage capacity. As with many of these studies, these issues were identified primarily by researchers through a variety of interactions with the parents and children. In a follow-up study in Soweto in South Africa (8), feedback was sought directly from mothers. This included new insights that children's behaviour appeared unaffected by the presence of the WCs in the various situations in which they were used, that there was generally a high level of acceptance of the use of WCs in families, and that having clear visible indication of when WCs were recording was important to parents.

The findings from these studies illustrate the importance of directly seeking feedback from a range of stakeholders involved in this kind of research. In the current study, for the first time, we drew on the experiences of both parents, and the different types of researchers (academics, fieldworkers, video coders) contributing to our research using WCs to explore parent-child interactions. We collaborated with an industry partner specialising in health-related product design to identify and prioritise the challenges and issues faced by each of those groups of stakeholders. In addition to helping us understand the challenges themselves, this allowed us to build a set of requirements that; (i) enabled the identification of an alternative WC device already on the market that had the potential for immediate improvements (e.g., increased speed and accuracy of coding footage from WCs) and, (ii) will be of broad use to other researchers interested in using WCs to capture footage of interactions in free-living conditions.

## Methods

### Overview

We worked with Kinneir Dufort (KD), a user-centred digital product design consultancy based in Bristol, UK. We began by conducting an exercise capturing challenges around the use of WCs for researching parent-child interactions from a range of stakeholders. Participants were primarily from the research team and participants in the Avon Longitudinal Study of Parents and Children (ALSPAC) at the University of Bristol, UK, but also included input from research teams from SAMRC/Wits Developmental Pathways for Health Research Unit study at University of the Witwatersrand, South Africa. The outputs of this exercise were prioritised lists of challenges for each stakeholder group. The top priority challenges across the stakeholder groups were then used as criteria to select a new, improved WCs device. We identified one issue that had been particularly problematic in our experience with WCs, coding interactions when the faces of parents or children were not in view, and compared performance in this for the old and new WCs.

### Participants

Four types of stakeholders were identified, and participants of mixed age, sex, and backgrounds were invited to take part in sessions identifying challenges (numbers in parentheses are numbers that attended):

Parents (*n* = 3)

Researchers (*n* = 9):

Academics, conceptualising and designing the study (*n* = 5)*
Fieldworkers, working with participants to implement the study (*n* = 3)
Data processors, downloading, processing and coding the captured footage (*n* = 4)*

*Note: Three of the four Data processors were Academics who had extensive experience coding videos so also responded as Data processors.

In line with local ethical guidance, the small numbers in each group meant demographic data was not recorded to ensure anonymity.

### Video footage study cohort

All video footage used in the comparison of WCs in this study was recorded from participants from the ALSPAC cohort, and information on this cohort is presented below.

During Phase I enrolment, 14,541 pregnant mothers residing in the former Avon Health Authority in the south-west of England with expected dates of delivery between 1 April 1991 and 31 December 1992 were recruited. Of these initial pregnancies, there was a total of 14,676 foetuses, resulting in 14,062 live births and 13,988 children who were alive at 1 year of age. A further 913 pregnancies were recruited during Phases II, III and IV respectively, resulting in an additional 913 children being enrolled. The total sample size is 15,454 pregnancies, of which 14,901 were alive at 1 year of age. The Children-generation 2 (ALSPAC-G2) was set up to provide a unique multigenerational cohort and builds on the existing ALSPAC resource of originally recruited women and their partners (Generation 0; ALSPAC-G0) and their offspring (ALSPAC-G1) followed up for 26 years. Recruitment of the next generation ALSPAC-G2—the grandchildren of ALSPAC-G0 and children of ALSPAC-G1—began on 6th June 2012. Up to 30th June 2018, 810 ALSPAC-G2 participants from 548 families had been recruited. Over 70% of those invited to early- and late-pregnancy, second week of life, 6-, 12- and 24-month assessments attended, with attendance >60% for subsequent visits up to 7 years. Further details on the cohort profile, representativeness and phases of recruitment, including ALSPAC-G2, are described in four cohort-profile papers (11–14).

The ALSPAC study website www.bristol.ac.uk/alspac/ contains details of all the data that is available through a fully searchable data dictionary and variable search tool (http://www.bris.ac.uk/alspac/researchers/our-data/). Informed consent for the use of data collected via questionnaires and clinics was obtained from participants following the recommendations of the ALSPAC Ethics and Law Committee at the time.

Study data were collected and managed using REDCap electronic data capture tools hosted at the University of Bristol. REDCap (Research Electronic Data Capture) is a secure, web-based software platform designed to support data capture for research studies (15).

### Recruitment into the WCs study

Ethical approval for the study was obtained from the ALSPAC Ethics and Law Committee and the Local Research Ethics Committees. Recruitment of mothers into the headcams study began on 7th July 2016, with 422 (90%) of mothers and their infants attending a 6-months assessment at the research clinic. 266 (63%) of mothers who attended the clinic were invited to record interactions with their infant using the headcams at home. 141 (53%) of these mothers consented to participate and 104 (74%) mothers provided video footage of mother-infant interactions. Initially, biological fathers and mothers' partners were invited to participate in the headcams study indirectly through an invitation to the mother when their child joined ALSPAC-G2. On 22nd July 2019, through additional funding from Wellcome Trust, a separate research clinic for fathers was set-up (Focus on Fathers) inviting fathers directly to attend a range of assessments, including the head cams, when their G2 child was six months old. Overall, 283 fathers were invited to attend, with 154 (54%) fathers consenting to participate and 86 (30%) fathers providing video footage of father-infant interactions.

### Videorecording procedures using the WCs

**Figure 1:**
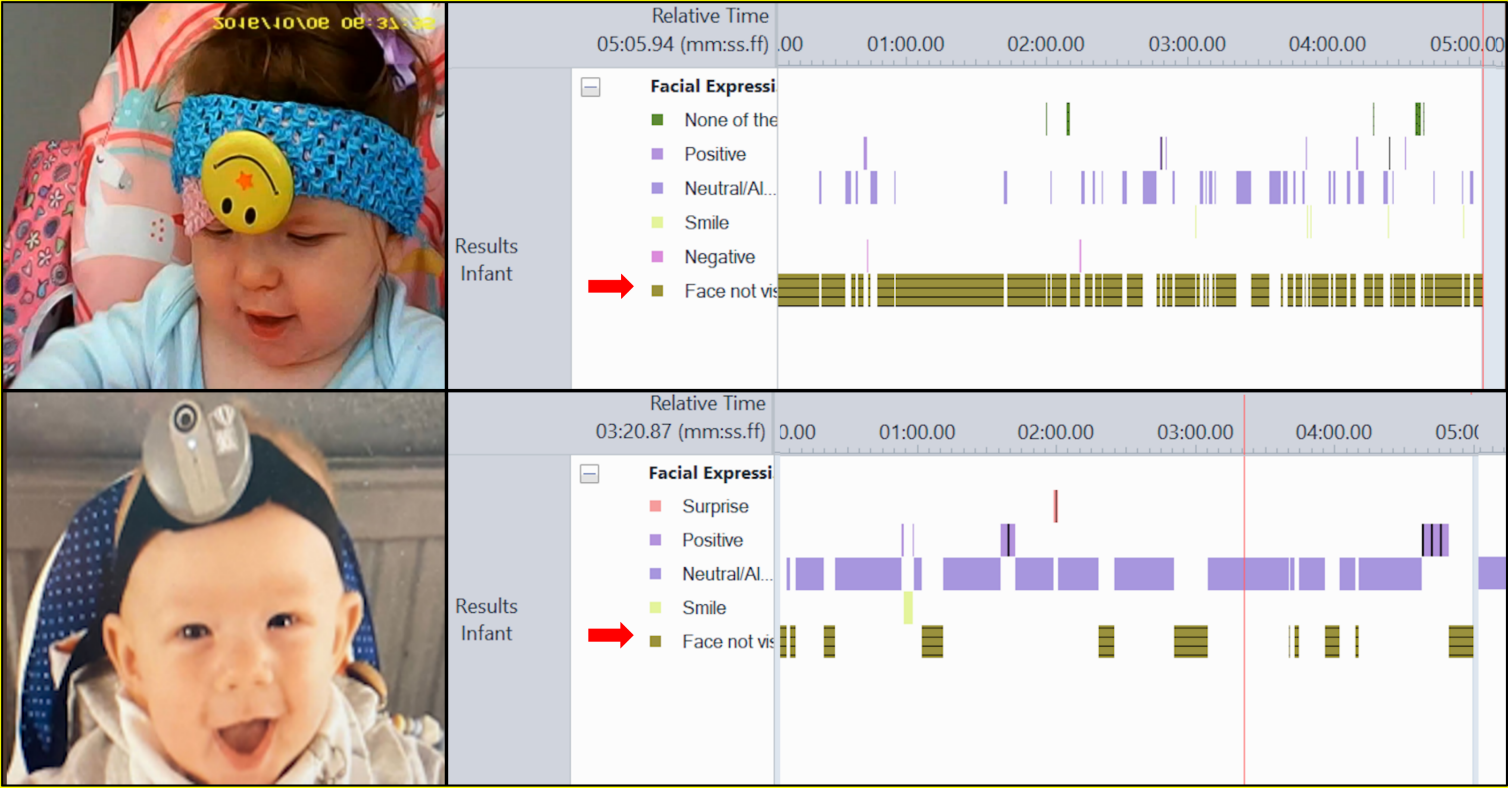
Old WCs (top left), and new WCs (bottom left). Micro-coding showing amount of time the observed face is not in view (highlighted with red arrows) for old WCs (top right) and new WCs (bottom right). Note micro-codings are examples for those devices and not for the infants shown.

We captured video and audio footage of mother-infant and father-infant interactions using the type of WC previously used to record infant's eye views of their environment (4). The WC was available in a variety of different specifications from many different manufacturers. We used devices from Boddban, which had a resolution of 720 × 480 pixels at 30 frames/s, and a field of view of approximately 60 degrees (referred to here as the “old” WCs device and shown in Figure 1). The WCs were worn on headbands by both the parent (mother or father) and the infant, capturing two separate videos from the parent and infant perspective for each interaction. WCs protocols were identical for both mothers and fathers. Parents were given fully-charged WCs and asked to use them at home during mealtime and play interactions. The separate WCs footage from the parent and infant cameras were subsequently synchronised by the researchers and interactions between the parent and infant were then coded using a micro-behavioural observation coding system developed by the project team (16).

### Procedures

#### Capturing stakeholder challenges

**Figure 2:**
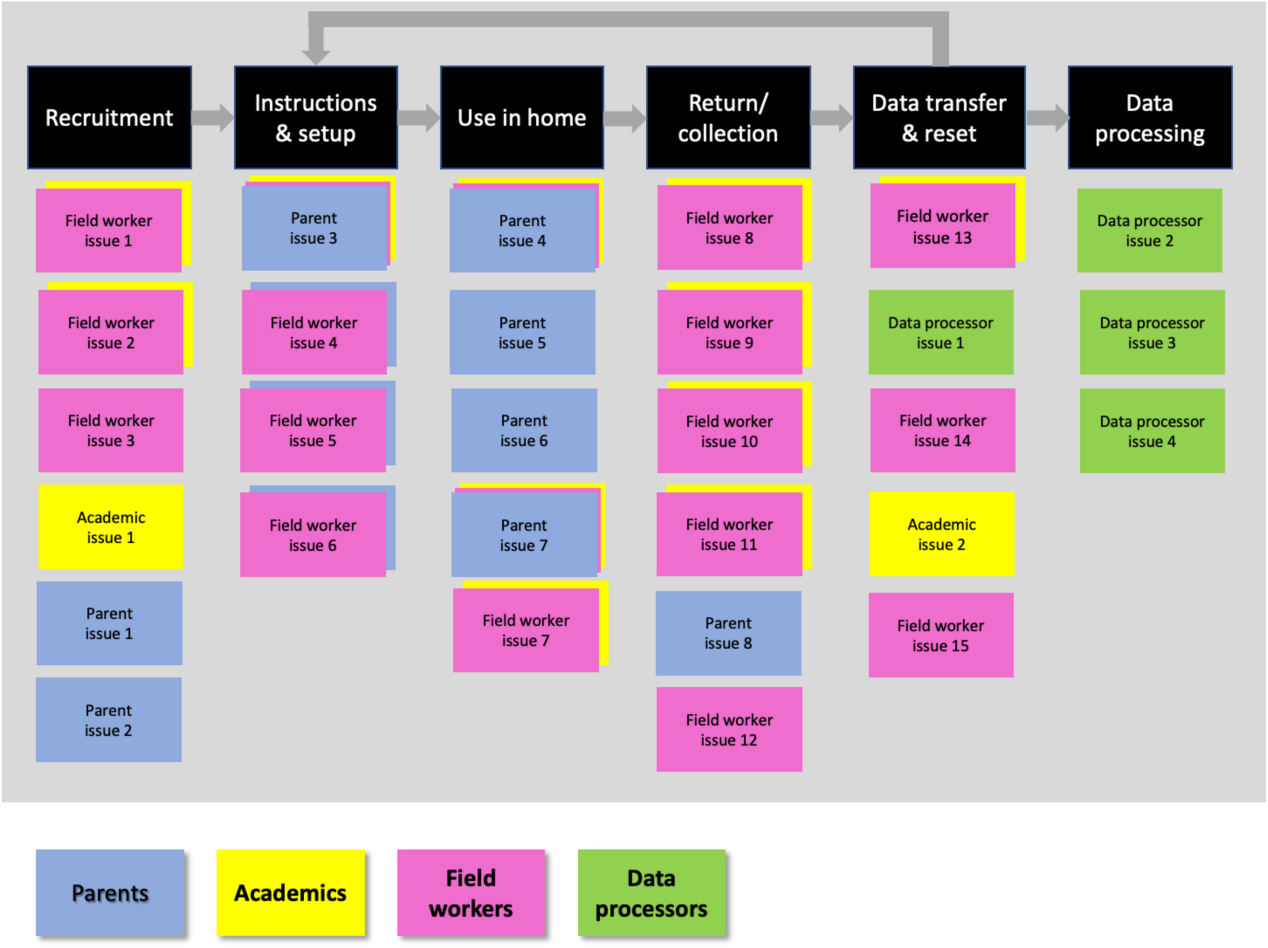
Illustration of method in which different stakeholders added challenges against study pipeline stages.

Two sessions were conducted in 2020. The sessions were physically based in a meeting room at the University of Bristol, and a virtual equivalent of the setup developed using the Mural Visual Collaboration software tool was presented to the research team in University of the Witwatersrand, and to colleagues previously involved with the studies but now living in Chile. For the in-person sessions in Bristol the stages in the study pipeline were presented as columns on a whiteboard. Each attendee was assigned to one of the stakeholder types, and each stakeholder type had a different colour card, so that while the challenges raised would remain anonymous, they could be grouped by stakeholder type (note that three academics also had extensive experience coding videos so also contributed as data processors). An illustration of the layout used is in shown in Figure 2. Attendees in the remote version of this sessions followed the same procedure with the same layout in Mural. Cards from each stakeholder group were combined, thematically similar challenges were grouped, and those raised most frequently were ranked as highest priority. Given the novelty and potentially intrusive nature of these new WCs methods, the attitudes and opinions of parents were of particular interest, so for this group we report additional detail on the challenges they identified.

#### Comparing old and new WCs devices

When reflecting on the various stages in our WCs studies, the most resource intensive step has been the coding of parent-child interactions in the WCs footage (7), and this echoed the findings of other recent reviews of WCs methods (6, 10) The main difficulty identified by the researchers was coding parent-child interactions when their faces were not in view. Having faces visible is particularly important when coding interactions so that facial expressions, and then emotional states can be coded accurately. Given this, we selected time “face not visible” as a measure to use to compare the performance of the old and new WCs. To quantify how the old and new WCs influenced this, whenever the face of a child or parent went out of frame we coded it as “face not visible” using the coding scheme developed by the team (16), and recorded the duration it was not visible (see Figure 1). We did this for all videos from the ALSPAC study that had 5 min of headcam footage from both a parent (mothers only) and child. 75 videos were processed in total (49 using the old WCs and 26 using the new WCs).

## Results

### Prioritised stakeholder challenges

**Figure 3:**
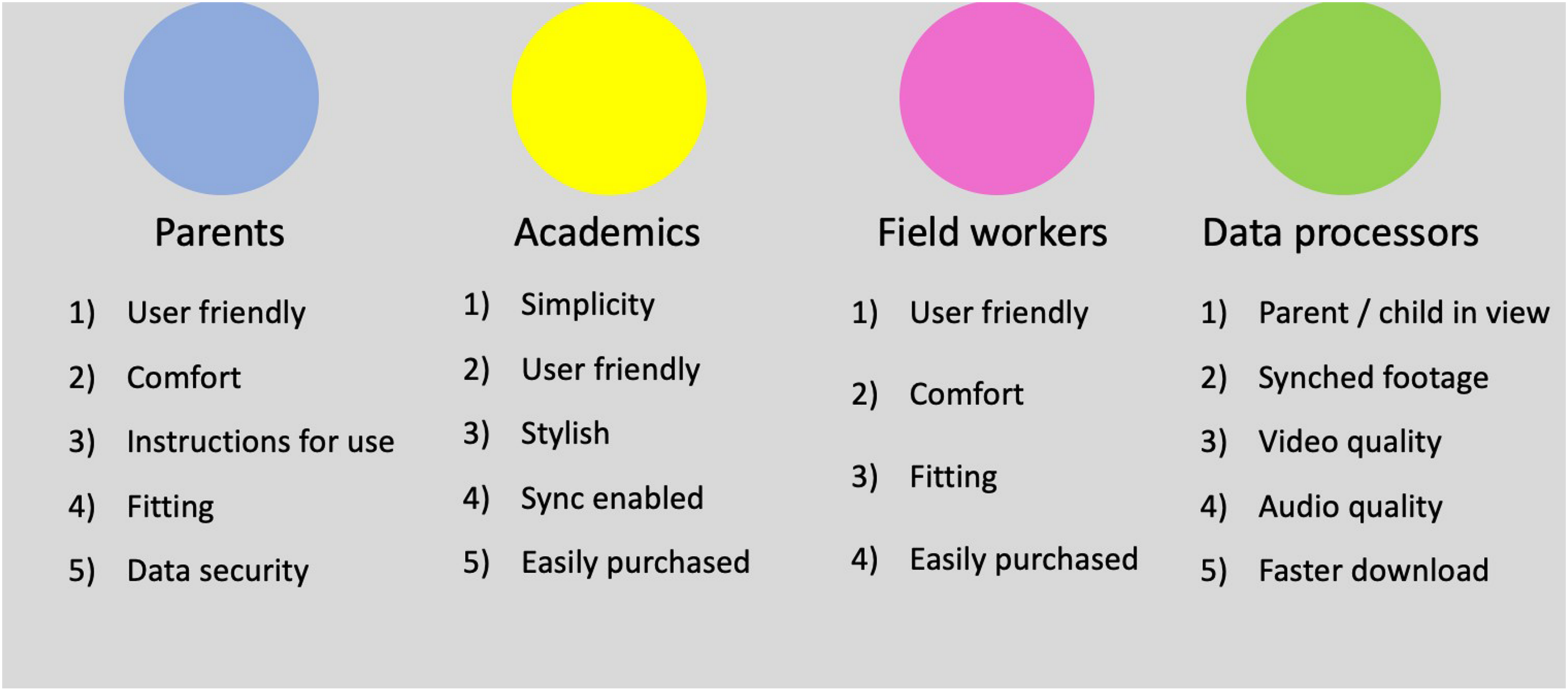
Prioritised lists of WCs challenges by stakeholder group.

The prioritised lists of challenges from each stakeholder group are shown in Figure 3.

### Key challenges and implications

Considering the top three priorities across the four stakeholder groups, there were five distinct challenges. These key challenges, with a summary of their implications (also captured from the cards in the two stakeholder sessions), are summarised in Table 1.

**Table 1:** Key challenges and implications.

Key Challenges	Implications
Simplicity and ease of use	Barriers to participant recruitment Higher attrition during study Excessive fieldworker time needed
Comfort and stability of fitting	Higher attrition during study Excessive fieldworker time needed Difficulties and delays coding interactions (faces and bodies of participants not in frame if WCs moves from optimal position)
Professional and stylish appearance	Barriers to participant recruitment
Camera field of view and video quality	Difficulties and delays coding interactions (faces and bodies of participants not in frame if WCs moves from optimal position)
Video footage format for synchronisation	Additional steps required processing footage before coding of interaction (e.g. if video stored in segments which need assembling into single sequence)

### Additional details from parents

To provide further detail on parents' issues and perspectives, for each of the priority challenges identified by the parents we have included a number of the most informative direct quotes from the parents in Table 2.

**Table 2:** Direct quotes from parents.

Priority Challenges	Quotes from parents
User friendly	“What does the light mean!? Wore all the time as a consequence.” “Having a box to keep it safe when not in use would be good.” “I found the orientation of the camera (using it upside down) confusing.”
Comfort	“Could camera be attached to a hat or cap instead?” “Of the potential new headbands I prefer the design without a securing band over the top of the head.” “It's reassuring to know the camera has been cleaned before I get it.”
Instructions for use	“Having a user guide to refer to would be useful.” “Would be helpful to know how long a charge lasts for.” “Had no idea how long it would take to charge.”
Fitting	“Adjustable headband to accommodate different size heads would be useful.” “Children try to remove cameras from themselves and parents.”
Data Security	“I want reassurance about exactly what the data will be used for.” “If the footage capture will be linked to mental health data in any way this needs to be clear in the information provided.” “What happens if the person coding the video knows me?”

### Candidate new WCs devices and performance against selection criteria

**Figure 4:**
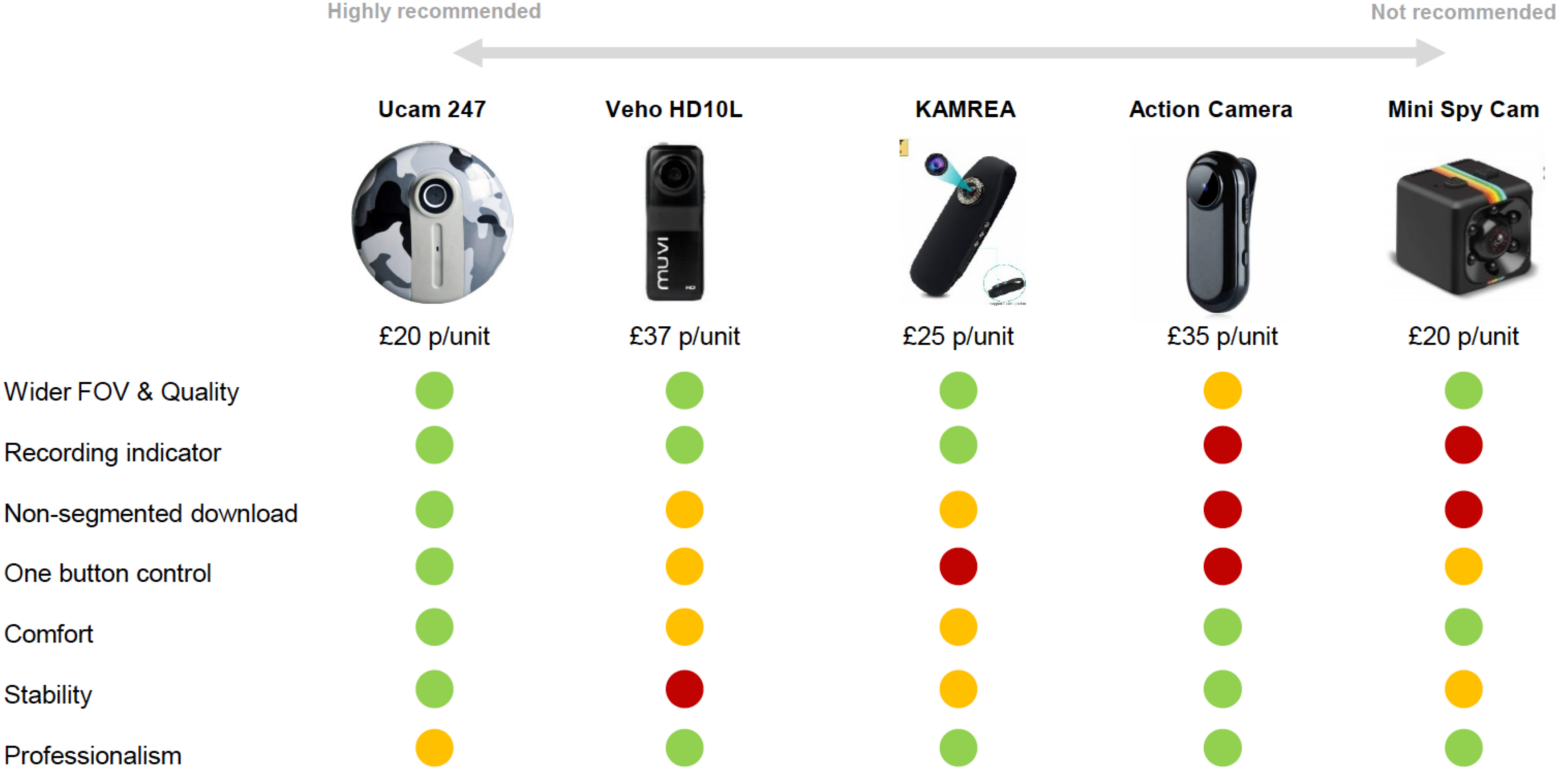
Candidate devices and performance against selection criteria.

The five devices identified as candidate new WCs (at the time of the work in 2020) and their performance against the selection criteria are shown in Figure 4. The device selected as the new WC to be trialled was the WearCam manufactured by Ucam 247 (see device in use in Figure 1). This had a resolution of 1280 × 720 30 frames/s and a field of view of 85 degrees.

### Comparisons of old and new WCs devices in key data processing stages

The data illustrating the median duration in seconds that the face was coded as “face not visible” are shown in Table 3. These statistics were derived using The Observer XT (v16) software package from Noldus (https://www.noldus.com/observer-xt) for coding behaviours in video footage, which uses an event-based, time stamped coding system to record the onset and offset of behaviours, which includes faces going out of view. The onset and offset times were used by The Observer XT package to compute the total number of “face not visible” occurrences and the duration of each occurrence, which were then combined to provide total durations per video (16).

**Table 3:** Face not visible duration per coded video.

WCs device	Median (sec)	Inter-quartile range (sec)	Upper quartile (sec)	Lower quartile (sec)
Old (19 parents, 30 infants)	16.3	25.9	33.9	8.1
New (12 parents, 14 infants)	4.7	3.1	5.7	2.7

## Discussions

### Principle findings

A set of five key challenges were identified across the different groups of stakeholders involved in research using WCs for measuring parent-child interactions in the UK cohort study ALSPAC, and SAMRC/Wits Developmental Pathways for Health Research Unit study at University of the Witwatersrand in South Africa:

• Simplicity and ease of use
• Comfort and stability of fitting
• Professional and stylish appearance
• Camera field of view and video quality
• Video footage format for synchronisation

Using these issues as selection criteria, it was possible to review a range of candidate WCs and select a new device potentially better suited to the specific needs of recording parent-child interactions.

To compare the performance of the old and newly selected WCs, we identified time “face not visible” per video as a key downstream metric, because having the faces of the parent and child visible is vital in the coding of emotional state and reactions needed to assess parent-child interactions. The median “face not visible” time per video for the new WC was reduced to approximately a quarter that of the old device. This improvement in keeping faces of parents and children in view, most likely the result of a combination of increased field of view, video quality (e.g., adaptation to varying light levels), ease of use, and secure fitting, will considerably reduce the likelihood of missing a reaction or a change in facial expression, making coding easier and faster.

### Key recommendations for future research

From the challenges identified across the stakeholder groups in this study, we make the following suggestions for future research using WCs.

When selecting a WC, ensure:

• The device has simple to use controls, ideally a single button for starting and stopping recording.
• The device has a clear visual indicator showing when it is recording.
• The device has age-appropriate fixing mechanisms that enable the device to be located stably in an optimal position, and that are comfortable for the participant (particularly important if the participant is a young child).
• The device has a professional appearance.
• The field of view of the device is wide enough to capture the events of interest, taking into account younger children are likely to have higher levels of head movement.
• The device is sufficiently sensitive to work in (reasonably) low light conditions inside homes.
• The device stores video footage in a format that simplifies video synchronisation across multiple cameras (e.g. make sure footage is not stored as multiple segments).
Once the device is selected, simple to follow instructions are developed and tested with parents.

### Strengths and weaknesses

To the best of our knowledge, this is the first study to have directly sought feedback from both parents and researchers in a range of different roles (academic, fieldworker, and data processors) about the use of WCs to study parent-child interactions. The varied stakeholders contributing to this study were drawn from what is potentially the largest study to date using WCs to assess parent-child interactions. The fieldworkers, researchers and data processors in particular have considerable experience recruiting and engaging families in research, working with parent and child dyads, processing WCs footage, and coding parent-child interactions.

The novel and potentially intrusive nature of these new WCs methods means the opinions of parents were of particular interest, and we have included additional information from parents in the form of direct quotes. Future studies of this topic would benefit from more extensive qualitative research with parents. While the nature of the cohort studies in which this work was conducted meant the parents were well matched for age, variations in other factors, including social-economic position may have influenced the use of the WCs and the comparison of device performance reported here.

The candidate new WC devices considered and the device selected will have been specific to the time the study was conducted (2020), and will likely have been superseded by the time of publication. However, the challenges identified in the use of WCs for measuring parent-child interactions, and the approach for using these to consider and select new WCs will remain relevant over time.

## Conclusions

Working with a range of stakeholders involved in projects utilising WCs to assess parent-child interactions, and an industry partner specialising in digital product design, we identified a number of priority challenges relating to use of WCs in this field of research. These issues were then used as selection criteria to identify a new WC device optimally suited to capturing footage of parent-child interactions. This new device dramatically improved one of the key factors enabling quality coding of interactions—the extent to which the faces of all of the individuals interacting are in view in the footage captured. The challenges identified will be important in guiding future research using WCs to assess interaction between parents and children, and more broadly in the use of WCs in advancing methods for objective assessments in a range of health research domains (17).

## Data Availability

The original contributions presented in the study are included in the article/**Supplementary Material**, further inquiries can be directed to the corresponding author/s.
