# Supplementary material for: Identifying stakeholder priorities in use of wearable cameras for researching parent-child interactions
Source: Front Child Adolesc Psychiatry. 2023 Jun 23;2:1111299. doi: 10.3389/frcha.2023.1111299 (PMC7616607; doi:10.3389/frcha.2023.1111299)
Supplement: Supplementary file 1 [file Datasheet1.docx]

**Supplementary Materials**

*Removing the need for a researcher to be present*

When attempting to study natural interactions between people in free-living conditions having a researcher present can considerably reduce the ecological validity of a study. This is particularly true when studying parent-infant interactions, when the presence of a researcher can be significant distraction for the infant, as is illustrated in Figure S1.


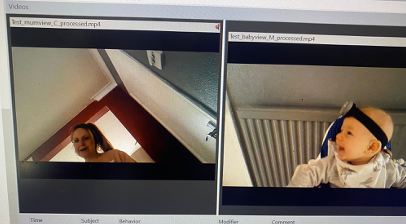


Figure S1. Impact of researcher presence when studying parent-infant interactions. Right: image from parent’s camera. Left: image from infant’s camera oriented towards researcher, with parent not in frame.

*Impact of face not visible on coding of expressions*

The impact of faces not being visible is further illustrated by the example in Figure S2, with only 2 seconds of the face not visible the sequence of negative followed by neutral expression is still likely captured, but the exact time point at which the infant transitioned from mild distress to neutral (potentially important in understanding certain interactions) is lost.


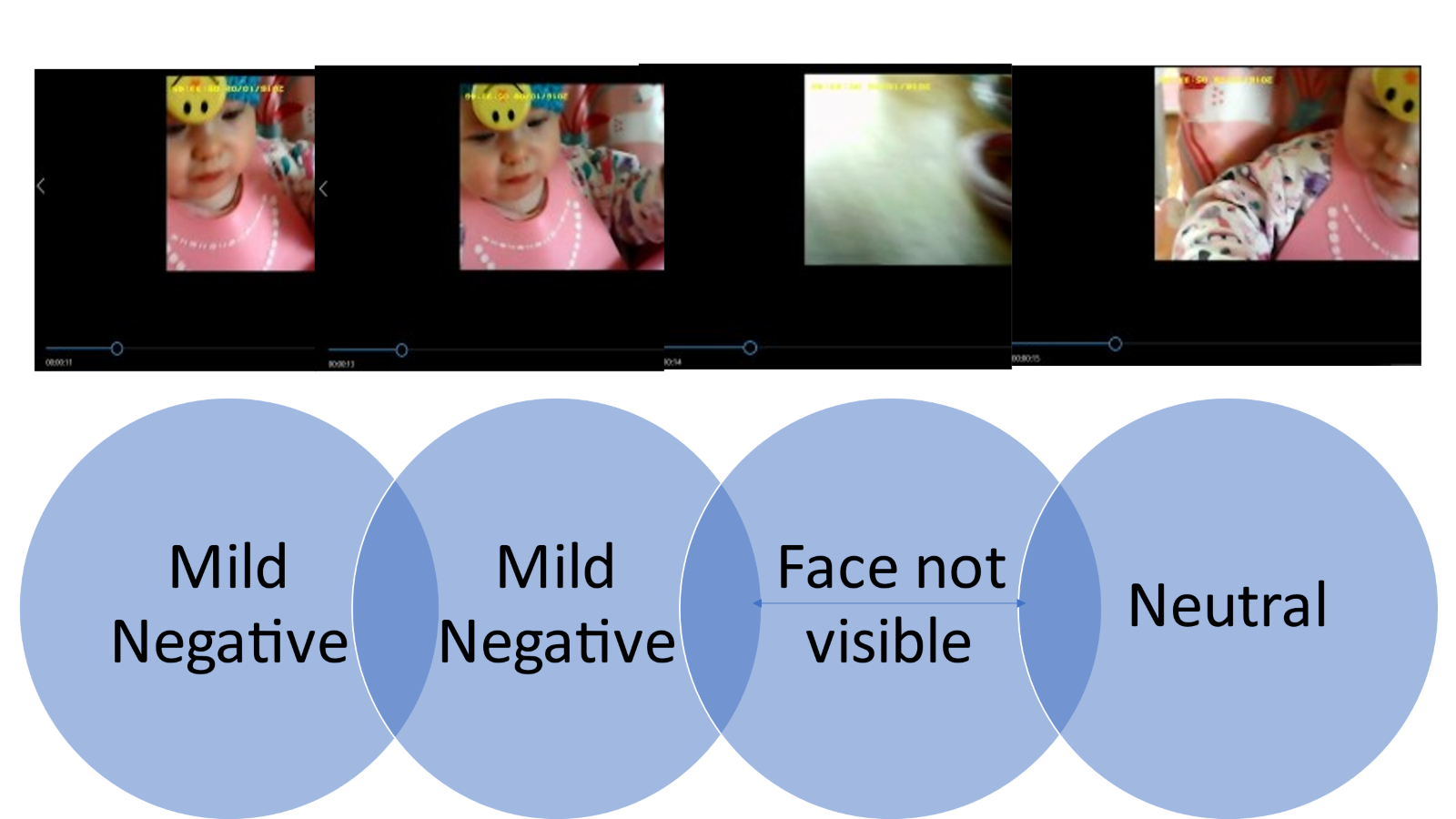


Figure S2. Impact of face not visible on coding of facial expressions
