# Supplementary material for: Identifying stakeholder priorities in use of wearable cameras for researching parent-child interactions
Source: Front Child Adolesc Psychiatry. Author manuscript; Available in PMC 2024 Sep 20. (PMC7616607; doi:10.3389/frcha.2023.1111299)
Supplement: Supplementary file 1 [file EMS198061-supplement-Supplementary_material.pdf]

Supplementary material

The Supplementary Material for this article can be found online at: https://www.frontiersin.org/articles/10.3389/frcha.2023.1111299/full#supplementary-material.
